# Development and Psychometric Testing of the Inventory of Self‐Care Decision‐Making Styles of Older Adults

**DOI:** 10.1002/nur.70031

**Published:** 2025-11-18

**Authors:** Maria Matarese, Marzia Lommi, Maddalena De Maria, Barbara Riegel

**Affiliations:** ^1^ Department of Medicine and Surgery Campus Bio‐Medico University Rome Italy; ^2^ Department of Clinical and Molecular Medicine, School of Nursing, Faculty of Medicine and Psychology University La Sapienza Rome Italy; ^3^ Department of Life Science, Health and Health Professions Link Campus University Rome Italy; ^4^ University of Pennsylvania School of Nursing Philadelphia Pennsylvania USA; ^5^ Center for Home Care Policy & Research at VNS Health New York New York USA

**Keywords:** decision making, instrument development, older adults, psychometrics, self‐care

## Abstract

Understanding how older adults make decisions about their self‐care is essential for designing tailored interventions that promote autonomy and well‐being. However, no instrument currently exists to specifically measure self‐care decision‐making styles in this population. The study aimed to develop and test the psychometric properties of the Self‐Care Decision‐Making Styles of Older Adult (DECIDE) Inventory, a new instrument designed to assess styles of self‐care decision‐making among older adults. A three‐phase process was undertaken: (i) instrument development based on theory and previous studies; (ii) content validity evaluation with 10 experts and cognitive interviews with 10 older adults; and (iii) psychometric testing, including assessment of structural and construct validity, internal consistency, and test‐retest reliability. A convenience sample of 350 older adults from various Italian regions participated in the validation phase. The study followed the COnsensus‐based Standards for the selection of health Measurement INstruments (COSMIN) reporting guidelines. Following content validation, an instrument was finalized, assessing four decision‐making styles—independent, responsible, self‐neglected, and guided—, with five items per scale. Confirmatory factor analyses supported the structural validity of the four‐scale model. Construct validity was confirmed through hypothesis testing. Reliability indicators, including Omega coefficients, factor score determinacy, item‐total correlations, and intraclass correlation coefficients, ranged from adequate to optimal across all scales. The Self‐Care Decision‐Making Styles of Older Adult Inventory demonstrated good validity and adequate reliability for assessing self‐care decision‐making styles in older adults. Further refinement and cross‐cultural validation are recommended before its use in clinical practice.

## 1 Introduction

The population aged 65 and over is increasing worldwide. In Europe, it is projected to grow from 90.5 million to 129.8 million by 2050, with the largest rise expected among those aged 75–84 years (European Commission Eurostat 2020). As a result, healthcare services are increasingly focused on helping older adults remain at home, preserving their identity, autonomy, and quality of life (Canadian Agency for Drugs and Technologies in Health 2024; World Health Organization 2015). Achieving these goals requires promoting effective self‐care, which is essential for managing health and chronic conditions (Mielenz et al. 2021; Wong et al. 2019). The Middle‐Range Theory for Self‐Care of Home‐Dwelling Elderly conceptualizes self‐care as a partly conscious and partly subconscious adaptation to age‐related changes (Backman and Hentinen 1999; Räsänen et al. 2007). Older adults' self‐care practices reflect their personal histories, personalities, health status, and attitudes towards aging and the future, expressed through four distinct self‐care types: independent, responsible, formally guided, and abandoned (Backman and Hentinen 1999). Independent self‐care involves managing health and daily activities based on personal beliefs and experience, valuing autonomy over healthcare professionals' (HCPs) recommendations. Responsible self‐care entails actively maintaining health, understanding treatments, collaborating with HCPs, and maintaining a positive outlook on aging and future. Formally guided self‐care is characterized by strict adherence to medical advice and structured routines, reflecting pragmatic acceptance of aging. Abandoned self‐care involves dependency and disengagement, due to impairments or negative life experiences (Backman and Hentinen 1999).

Self‐care relies on a naturalistic decision‐making process involving need recognition, information gathering, option evaluation, choice making, and outcome evaluation in complex real‐world situations (Riegel et al. 2012). This process is shaped by decision‐making styles, or the habitual patterns individuals use when making decisions (Danya and Nakayama 2022). Individuals typically follow a dominant decision‐making style, although different situations may prompt a shift (Scott and Bruce 1995). Each self‐care type described by Backman and Hentinen (1999) can reflect a distinct decision‐making style, influencing each phase of the process. For example, during the information‐gathering, a responsible type might thoroughly research information about a health issue consulting multiple sources, while an independent type might rely on personal experience and intuition. In option evaluation, abandoned self‐care type might disengage, whereas formally guided type may defer to HCPs. To date, no instrument exists to directly assess self‐care decision‐making styles in older adults. The only available instrument, the Self‐Care of Home‐Dwelling Elderly (SCHDE) scale, focuses on measuring self‐care behaviors as defined by the four types proposed in the Middle‐Range Theory for Self‐Care of Home‐Dwelling Elderly (Räsänen et al. 2007; Räsänen et al. 2010). However, it does not capture the underlying decision‐making processes. Furthermore, several studies have reported inconsistent findings regarding the SCHDE factorial structure, indicating that its structural validity remains uncertain (Evaristo and Marques 2018; Imaginário et al. 2020; Räsänen et al. 2007; Räsänen et al. 2010; Železnik 2010).

Identifying decision‐making styles can clarify the cognitive drivers of self‐care behaviors and highlight barriers and facilitators missed by behavior‐focused tools. This knowledge can support nurses in tailoring interventions that promote independence and aging in place.

The aim of this study was twofold: (i) to develop the Self‐care Decision making Styles of Older People (DECIDE) Inventory to measure decision‐making styles in self‐care among older adults, and (ii) to evaluate its psychometric properties.

## 2 Methods

### 2.1 Study Design

A three‐phase process was followed: i) instrument development; ii) content validity assessment, and iii) psychometric testing, including evaluations of structural validity, construct validity, internal consistency, and test‐retest reliability. The study adhered to the COSMIN (COnsensus‐based Standards for the selection of health Measurement INstruments) guidelines for reporting (Gagnier et al. 2021; Terwee et al. 2018) (Supporting Information S1: Table 1).

### 2.2 Instrument Development

Although Klein's (2008) work on naturalistic decision‐making provides valuable insights into how decisions are made in real‐world, high‐pressure situations, it does not conceptualize distinct decision‐making styles, which was the focus of our study. For this reason, we based instrument development on Thunholm's (2004) conceptual framework of decision‐making styles. Thunholm conceptualizes decision‐making style as a multidimensional construct encompassing information processing, self‐evaluation, and self‐regulation, shaped not only by habits and situational or task‐specific demands but also by enduring individual characteristics. This comprehensive perspective provides a strong foundation for examining real‐world choices typically made under uncertainty, time pressure, and high complexity, such as those encountered in self‐care (Thunholm 2004).

Item generation was also informed by the four self‐care types proposed in the Middle‐Range Theory for Self‐Care of Home‐Dwelling Elderly (Backman and Hentinen 1999). The research team identified relevant concepts from both sources and developed an initial pool of items reflecting decision‐making processes related to self‐care in older adults. This pool was refined through an iterative, consensus‐driven process in which the research team members reviewed, discussed, and modified items until agreement was reached on a final set of 30 items. These items were organized into four distinct self‐care decision‐making style scales. The Responsible Self‐Care Decision‐Making Style Scale—hereafter referred to as the Responsible Decisions Scale—was represented by eight items (e.g., I try to gather all useful information before making decisions about an illness). The Independent Self‐Care Decision‐Making Style Scale—hereafter referred to as the Independent Decisions Scale—included seven items (e.g., I decide to use my own methods to solve a health problem rather than seeking help). The Guided Self‐Care Decision‐Making Style Scale—hereafter referred to as the Guided Decisions Scale—was illustrated by seven items (e.g., I completely rely on healthcare personnel when I have to make a decision about an illness without trying to solve it on my own). The Self‐Neglected Self‐Care Decision‐Making Style Scale—hereafter referred to as the Self‐Neglected Decisions Scale—was measured by eight items (e.g., I do not care who makes decisions about my health). A 5‐point Likert scale, ranging from strongly disagree (1) to strongly agree (5), was used to balance discrimination with cognitive load in older adult populations (Koo and Yang 2025). Each participant's dominant self‐care decision style was identified by averaging item scores within each style; the highest score indicated their prevailing decision‐making style.

### 2.3 Content Validity

Content validity was assessed by evaluating the relevance and comprehensiveness of the items through a review of 10 experts: two clinical nurses specialized in geriatric care, four nursing professors, and four nurse experts in self‐care. After an introductory explanation of the four self‐care decision‐making styles, the experts completed an online survey in which they rated each item's relevance on a 4‐point scale: 1 = not relevant, 2 = somewhat relevant, 3 = quite relevant, and 4 = very relevant. The Item Content Validity Ratio (I‐CVR) for each item was calculated using the formula (Ne‐N/2)/(N/2), where Ne represents the number of experts rating the item as quite or very relevant (3 or 4), and N is the total number of experts (Lawshe 1975). I‐CVR values range from ‐1 to 1, with a critical threshold of 0.80 suggested for a panel of 10 experts to indicate acceptable agreement beyond chance (Ayre and Scally 2014). Experts were also invited to suggest additional items to improve the instrument's comprehensiveness. The I‐CVR values ranged from 0.50 to 1.00. Ten items with I‐CVR scores below the 0.80 threshold were removed (see Supporting Information S1: Table 2). The removal of these items did not compromise coverage of the core decision‐making processes within each style. The overall Scale Content Validity Index (S‐CVR), calculated as the mean of the remaining 20 I‐CVR values, (Ayre and Scally 2014), was 0.85. Two researchers independently reviewed the experts' suggestions, but no additional items were deemed necessary. Therefore, the inventory resulted in 20 items in total, five items for each decision style scale. To assess item clarity and comprehensibility, cognitive interviews were conducted with 10 older adults (six women, four men, aged 70–87 years) recruited from a day center. Participants, after providing informed consent, first completed the instrument independently, followed by a focused interview exploring their understanding of the items and instructions. Interviews were audio‐recorded, transcribed, and analyzed by the research team. Based on these interviews, minor revisions were made to enhance clarity, including reordering clauses in some items, replacing “illness” with “health issue,” and adding the statement “There are no right or wrong answers” to reduce socially desirable responding.

### 2.4 Instrument Validity and Reliability Testing

Structural validity, construct validity, internal consistency, and test‐retest reliability of the 20‐item DECIDE inventory were assessed through a cross‐sectional study.

#### 2.4.1 Sample

A convenience sample of older adults was recruited through the personal networks of master's nursing students involved in data collection. Eligible participants were aged 65 years or older and living with any health condition. Exclusion criteria included cognitive or visual impairments affecting reading comprehension, insufficient proficiency in Italian, or unwillingness to provide informed consent. A minimum sample of 200 participants was required to test the instrument's dimensionality and internal consistency, based on the recommended ratio of 10 participants per item (De Vet et al. 2011).

#### 2.4.2 Data Collection

To ensure consistency, the master's nursing students received training from a senior researcher (ML), and data collection formed part of their coursework. Their participation was voluntary and did not influence students' academic evaluation. They approached potential participants, provided detailed explanations regarding the study's aims, obtained written informed consent, and administered paper‐based questionnaires.

While Thunholm's (2004) framework informed the conceptualization and item development through its comprehensive definition of decision‐making style, the construct validity testing of the instrument was grounded in the Middle‐Range Theory for Self‐Care of Home‐Dwelling Elderly (Backman and Hentinen 1999). This choice reflected our aim to situate decision‐making styles within the broader context of self‐care behaviors. Earlier studies showed that responsible and formally guided self‐care types were linked to higher functional autonomy (i.e., the ability to independently perform daily activities), life satisfaction, and self‐esteem, whereas the abandoned type correlated with lower levels of these variables; the independent type was linked to higher functional autonomy and self‐esteem (Backman and Hentinen 2001; Imaginário et al. 2020; Räsänen et al. 2014; Železnik 2010). Additionally, independent and responsible types correlated with an internal locus of control, while abandoned type correlates with an external one (Räsänen et al. 2010). Research on self‐efficacy—the confidence on one's ability to overcome difficulties when performing specific tasks—has also demonstrated that higher self‐efficacy promotes better self‐care behaviors (Wang et al. 2024).

Based on this evidence, it was hypothesized that the Responsible Decisions Scale would exhibit low to moderate positive correlations with functional autonomy, life satisfaction, self‐esteem, internal locus of control, and self‐efficacy, while the Self‐Neglected Decisions Scale would correlate negatively with these. The Independent Decisions Scale was expected to correlate positively, from low to moderate, with functional autonomy, self‐esteem, and internal locus of control, and the Guided Decisions Scale with functional autonomy, self‐esteem, and life satisfaction.

To assess criterion validity, associations between decision‐making styles and self‐care behaviors were examined. It was hypothesized that older adults exhibiting a responsible decisions style would demonstrate higher levels of self‐care, while those with self‐neglected, guided, and independent decisions styles would report comparatively lower levels of self‐care (Backman and Hentinen 1999; Riegel et al. 2012).

#### 2.4.3 Measures

Functional autonomy was assessed with the 8‐item Instrumental Activities of Daily Living (IADL) Index (Lawton and Brody 1969), a self‐report tool scored from 0 to 8, with higher score indicating greater functional autonomy in daily social activities.

Self‐efficacy was measured using the 10‐item General Self‐Efficacy Scale (GSES) (Schwarzer and Jerusalem 1995), in the validated Italian version by Sibilia et al. (1995). Items are rated from 1 (not at all true) to 4 (completely true) with higher scores reflecting greater self‐efficacy.

Life satisfaction was evaluated with the 5‐item Satisfaction With Life Scale (SWLS) (Diener et al. 1985), using the Italian validated version by Di Fabio and Gori (2016). Responses range from 1 (strongly disagree) to 7 (strongly agree), with higher scores indicating greater life satisfaction.

Locus of control, defined as the extent to which individuals perceive life events as determined by personal or external factors, was assessed with the 17‐item Locus of Control Behavior Scale (LCBS) (Craig et al. 1984), in the Italian validated version by Farma and Cortivonis (2000). Items are rated from 0 (completely disagree) to 5 (completely agree), with higher scores indicating greater external locus of control.

Self‐esteem was measured with the 10‐item Rosenberg Self‐esteem Scale (RSES) (Rosenberg 1965), in the Italian validated version by Prezza et al. (1997). Items are rated from 0 (strongly agree) to 3 (strongly disagree), with higher scores reflecting greater self‐esteem.

Self‐care behaviors were assessed with the 20‐item Self‐Care Inventory (SCI), which has demonstrated optimal reliability and validity in Italian older adults (Luciani et al. 2022; Luciani et al. 2025). The instrument comprises three scales: 8‐item Self‐Care Maintenance that includes behaviors carried out to maintain physical and emotional stability; 5‐item Self‐Care Monitoring that entails the process of observing oneself for changes in health status; and 6‐item Self‐Care Management that comprises the actions taken in response to symptoms or changes in health. Each item is rated from 1 (never/not likely) to 5 (always/very likely). For each scale, scores are standardized on a 0–100 scale, with higher values indicating higher self‐care.

A sociodemographic questionnaire was used to collect data on age, sex assigned at birth, perceived economic status (less than needed for living, enough, more than needed), marital status (married, widowed, divorced, single), employment (employed, retired), living situation (alone, with family, in residential care facility), and education level ( ≤ 8 years, ≥ 9 years).

#### 2.4.4 Data Analysis

Descriptive statistics (mean, standard deviation [SD], frequency, percentage, kurtosis, and skewness) were used to describe the sample. Normality was assumed with skewness and kurtosis values ≤|2|. Outliers were identified using Mahalanobis distance (*p* < 0.001) (Tabachnick and Fidell 2013) and missing data patterns were checked for randomness. Structural validity was tested via Confirmatory Factor Analyses (CFA) using the robust maximum likelihood (ML‐R) estimator (Muthén and Muthén 1998–2017). Model fit was evaluated through a multifaceted approach (Tanaka 1993): Comparative Fit Index (CFI) and Tucker and Lewis index (TLI) with values 0.90–0.95 indicating acceptable fit, and > 0.95 good fit; Root Mean Square Error of Approximation (RMSEA) with values ≤ 0.05 indicating a well‐fitting model, 0.05–0.08 moderate fit, and ≥ 0.10 poor fit, with 90% confidence intervals (CI) ≤ 0.05 to ≤ 0.08 indicating good fit, and *p*‐values > 0.05 indicating good fit; and Standardized Root Mean Square Residual (SRMR) with values ≤ 0.08 indicating good fit (Hu and Bentler 1999). Chi‐square (χ^2^) statistics were reported and used to interpret model fit alongside the above indices (Kline 1986). Modification indices and residual covariances were considered when theoretically justified. Factor loadings ≥|0.30| were considered acceptable (Floyd and Widaman 1995). Because the four decision‐making styles represent distinct constructs, rather than facets of a single higher‐order factor, we did not conduct a CFA on the total inventory. Such analysis assume that all items reflect one or more common underlying factors, which did not align with the theoretical structure of the DECIDE inventory. Instead, separate CFAs were conducted for each subscale to confirm that the items within each scale coherently measured their specific construct, allowing for a valid assessment of the internal structure of each decision‐making style (Floyd and Widaman 1995).

Construct validity was assessed through hypothesis testing using bivariate Pearson's or Spearman correlations, as appropriated, and multiple linear regression analyses. Correlation values of 0.10–0.29, 0.30–0.49, and ≥ 0.50 were interpreted as small, moderate, and strong, respectively (Cohen 1988). Standard multiple regressions analyses, after checking for multicollinearity, were conducted to examine the unique contribution of each predictor (GSE, SWL, LCBS, SES, IADL) to each decisions style, controlling for the others. Additionally, linear regression analyses assessed the associations between each decisions style scale and the three dimensions of self‐care of the SCI. To account for variability in item loadings, factor scores from the decisions style scales were used in these analyses. Standardized regression coefficients (β) were interpreted according to the following cut‐off values: small effect (| β | = 0.10), moderate effect (| β | = 0.30), and large effect (| β | ≥ 0.50) (Cohen 1988; Funder and Ozer 2019).

Reliability was assessed through internal consistency and test‐retest stability. McDonald's Omega and factor score determinacy coefficients were preferred over Cronbach's alpha, since they offer more accurate estimates when factor loadings vary among items (McDonald 1999). Values ≥ 0.70 were considered acceptable. Item discrimination was estimated via corrected item‐total correlations (ITC), with values ≥ 0.30 deemed adequate (Kline 1986). Test‐retest reliability was assessed in a subsample of 50 older adults, randomly selected from the total sample, who completed the paper‐based questionnaires twice with a 1‐week interval between completion. The intraclass correlation coefficient (ICC) was calculated using a 2‐way random effects model for absolute agreement. A sample of 50 subjects was needed to detect an ICC of 0.80 (95% CI ± 0.1) with two measurements (De Vet et al. 2011). ICC values of 0.50–0.75, 0.75–0.90, and > 0.90 indicated moderate, good, and excellent reliability, respectively. Item‐level agreement was assessed using weighted Kappa (κ) with quadratic weights, with κ values of 0.41–0.60 considered moderate, 0.61–0.80 substantial, and 0.81–1.00 almost perfect (Landis and Koch 1977). All analyses were performed using SPSS version 29 for descriptive and reliability statistics, and Mplus for CFAs.

### 2.5 Ethical Consideration

The study was conducted in accordance with the principles of the Declaration of Helsinki (World Medical Association 2013). Ethical approval was obtained from a University Ethics Committee (No. 88/2021‐OSS). Informed consent was requested from all participants after providing information about the study. All data were collected and stored ensuring anonymity.

## 3 Results

### 3.1 Participants' Characteristics

A total of 350 older adults were recruited. Most were women (52.60%), with a mean age of 75.16 years (SD 7.36). The majority had ≤ 8 years of education (57.42%) and lived with family members (75.14%) (Table 1). Missing data accounted for 2% and were randomly distributed across the instruments. Descriptive statistics for the items of the decision‐making style scales are presented in Table 2.

**TABLE 1:** Characteristics of older adult sample (*n* = 350).

Variables	N (%)
Sex	
Female	184 (52.60)
Male	166 (47.40)
Education level	
≤ 8 years	201 (57.42)
≥ 9 years	149 (42.58)
Marital status	
Married	209 (59.71)
Widowed	96 (27.43)
Divorced	26 (7.71)
Single	18 (5.14)
Employment status	
Employed	57 (16.29)
Retired	293 (83.71)
Economic status	
Less than needed for living	13 (3.71)
Enough for living	249 (71.14)
More than needed for living	88 (25.14)
Living condition	
Alone	80 (22.86)
Family	263 (75.14)
Residential care facility	7 (2.00)
	**Mean (SD) [range]**
Age	75.16 (7.36) [65–98]
General Self‐Efficacy Scale	28.73 (5.62) [10–40]
Instrumental Activities of Daily Living Index	6.55 (1.94) [0–8]
Satisfaction With Life Scale	23.90 (6.42) [5–35]
Locus of control Behavior Scale	35.30 (11.50) [0–77]
Rosemberg Self‐esteem Scale	22.02 (4.55) [9–30]
Self‐Care Inventory	
Self‐Care Maintenance Scale	69.10 (16.36) [22–100]
Self‐Care Monitoring Scale	71.84 (21.54 [5–100]
Self‐Care Management Scale	63.68 (19.27) [13–100]

Abbreviation: SD, Standard Deviation.

**TABLE 2:** Descriptive statistics of the items of the self‐care decisions scales, loadings of factor solutions, and internal consistency (*n* = 350).

Items	Mean (SD)	Skewness	Kurtosis	Loading	
***Independent decisions scale***					
1) I make decisions about my health problems on my own, without needing to ask anyone for advice.	2.869 (1.383)	0.074	‐1.323	0.453	
5) When I have a health problem, I decide to use my own methods rather than seeking help.	2.429 (1.280)	0.420	‐1,033	0.796	
6) Even if healthcare personnel suggest a treatment for a health problem, I may decide not to follow it and do things my own way.	2.223 (1.340)	0.715	‐0.827	0.606	
9) I decide to take only the medications I think are useful for the health problems that matter to me.	2.823 (1.500)	0.167	‐1.446	0.415	
19) I make decisions about my health based on my own experiences and what has worked for me in the past.	3.200 (1.228)	‐0.303	‐0.956	0.480	
*Omega coefficient*					0.691
***Responsible decisions scale***					
2) When I need to decide about a health problem, I consult with healthcare personnel.	4.383 (0.841)	‐1.466	1.924	0.304	
4) I think carefully before making a decision about my health	3.871 (1.092)	‐0.900	0.158	0.558	
13) I try to find all the useful information I can before deciding what to do about a health problem	3.969 (1.106)	‐1.025	0.329	0.695	
15) When I have a health problem, I take all the time I need before deciding what to do, to be sure I have understood the advice from healthcare personnel.	3.769 (1.103)	‐0.705	‐0.253	0.662	
18) I consider all the possible options before deciding which action is most useful to address a health problem.	3.926 (1.010)	‐0.874	0.338	0.717	
*Omega coefficient*					0.732
***Self‐neglected decisions scale***					
3) I do not think it is important to make decisions about my health.	1.900 (1.217)	1.213	0.395	0.606	
7) I have decided not to take any responsibility for my health.	1.617 (1.031)	1.768	2.464	0.697	
10) I do not really care about making decisions about my health.	1.593 (0.983)	1.728	2.282	0.866	
12) I have decided not to follow any treatment recommended by healthcare personnel because I feel it is useless in my situation.	1.580 (1.067)	1.840	2.397	0.577	
17) I do not care who makes decisions about my health	1.606 (0.956)	1.415	0.785	0.539	
*Omega coefficient*					0.796
***Guided decisions scale***					
8) When I need to decide about a health problem, I fully rely on healthcare personnel, and I do not try to manage it by myself.	3.951 (1.105)	‐0.852	‐0.196	0.462	
11) When I need to decide about a health problem, I ask healthcare personnel, for advice because they know more than I do	4.400 (0.856)	‐1.558	2.373	0.315	
14) The decision about which medicines I need to take is up to my doctor without me needing to know what are for	3.571 (1.253)	‐0.581	‐0.695	0.669	
16) I prefer that healthcare personnel who know more than I do, make decisions about my health on my behalf.	3.529 (1.236)	‐0.592	‐0.655	0.651	
20) I follow my doctor's prescriptions and the advice of healthcare personnel exactly as given, without questioning or trying to understand the reasons.	3.720	1.154	0.130	0.658	
*Omega coefficient*					0.701

Abbreviation: SD, Standard Deviation.

### 3.2 Dimensionality and Reliability of the Self‐Care Decision‐Making Style Scales

#### 3.2.1 Independent Decisions Scale

*Dimensionality*. A one‐factor solution was hypothesized. CFA showed excellent fit: χ^2^(5, *N* = 350) = 4.286, *p* = 0.509; RMSEA = 0.000, 90% CI 0.000–0.069, *p* = 0.844; CFI = 1.000; TLI = 1.007; SRMR = 0.020 (Table 3). All factor loadings were statistically significant, ranging from 0.415 to 0.796 (Table 2).

**TABLE 3:** Final fit indices from confirmatory factor analysis for the self‐care decision making style scales (*n* = 350).

Self‐care decision‐making style scales	χ2	df	p(χ2)	CFI	TLI	SRMR	RMSEA (90% CI) *p* value
Independent	4.429	5	0.509	1.000	1.007	0.020	0.000 (0.000 0.069) 0.844
Responsible	3.429	5	0.634	1.000	1.015	0.017	0.000 (0.000 0.061) 0.902
Self‐Neglected	3.820	4	0.430	1.000	1.002	0.016	0.000 (0.000 0.079) 0.682
Guided	6.351	4	0.174	0.988	0.971	0.018	0.041 (0.000 0.098) 0.524

Abbreviations: CFI, Comparative Fit Index; CI, Confidence Interval; df, degree of freedom; *p*, *p* value; RMSEA, Root Mean Square Error of Approximation; SRMR, Standardized Root Mean Square Residual; TLI, Tucker and Lewis index; χ², Chi‐square.

*Reliability*. Internal consistency was acceptable (Omega = 0.691), with a factor score determinacy of 0.855. Item discrimination was adequate (ITCs 0.331–0.596). Test‐retest reliability was excellent (ICC = 0.946, 95% CI 0.995–0.969), with weighted κ values ranging from 0.774 to 0.871.

#### 3.2.2 Responsible Decisions Scale

*Dimensionality*. CFA confirmed a one‐factor solution with excellent fit: χ^2^(5, *N* = 350) = 3.429, *p* = 0.634; RMSEA = 0.000, 90% CI 0.000–0.061, *p* = 0.902; CFI = 1.000; TLI = 1.015; SRMR = 0.017 (Table 3). Factor loadings ranged from 0.304 to 0.717, all statistically significant (Table 2).

*Reliability*. Internal consistency was acceptable (Omega = 0.732), with a factor score determinacy of 0.839. ITCs ranged 0.301–0.591. Test‐retest reliability was excellent (ICC = 0.899, 95% CI 0.825–0.942). Weighted κ values ranged from 0.644 to 0.866.

#### 3.2.3 Self‐Neglected Decisions Scale

*Dimensionality*. Initial CFA showed a fair fit: χ^2^(5, *N* = 350) = 13.566, *p* = 0.192; RMSEA = 0.070, 90% CI 0.026–0.116, *p* = 0.192; CFI = 0.965; TLI = 0.929; SRMR = 0.020. Modification indices indicated excessive covariance between items #10 (“I do not really care about making decisions about my health”) and #17 (“I do not care who makes decisions about my health”). This covariance was theoretically justifiable, as both items reflect a general lack of engagement in health‐related decision‐making—one from the perspective of personal involvement (#10) and the other from willingness to defer decisions to others (#17). After specifying this, model fit improved: χ^2^(4, *N* = 350) = 3.820, *p* = 0.430; RMSEA = 0.000, 90% CI 0.000–0.079, *p* = 0.682; CFI = 1.000; TLI = 1.002; SRMR = 0.016 (Table 3). Factor loadings were statistically significant and ranged from 0.577 to 0.866 (Table 2).

*Reliability*. Omega coefficient was 0.796, with a factor score determinacy of 0.917. ITCs ranged 0.530–0.710. Test‐retest reliability was excellent (ICC = 0.970, 95% CI 0.947–0.983). Weighted κ values varied from 0.833 to 0.977.

#### 3.2.4 Guided Decisions Scale

*Dimensionality*. CFA on the five‐item one‐factor model initially showed poor fit: χ^2^(5, *N* = 350) = 47.224, *p* = 0.001; RMSEA = 0.155, 90% CI 0.117–0.197, *p* ≤ 0.001; CFI = 0.789; TLI = 0.578, SRMR = 0.074. Modification indices revealed high covariance between the residuals of items #11 (“When I need to decide about a health problem, I ask healthcare personnel for advice because they know more than I do”) and #8 (“When I need to decide about a health problem, I fully rely on healthcare personnel, and I do not try to manage it by myself”). This covariance was theoretically justifiable, as both items capture a general tendency to defer health‐related decisions to HCPs, with item #11 emphasizing seeking advice and item #8 emphasizing complete reliance on HCPs. The covariance was specified, resulting in improved model fit: χ^2^(4, *N* = 350) = 6.351, *p* = 0.174; RMSEA = 0.041, 90% CI 0.000–0.098, *p* ≤ 0.524; CFI = 0.988; TLI = 0.971; SRM = 0.018 (Table 3). Factor loadings ranged from 0.315 to 0.669, all significant (Table 2).

*Reliability*. Omega coefficient was 0.701, with a factor score determinacy of 0.849. ITCs ranged 0.384–0.524. Test‐retest reliability was optimal (ICC = 0.897, 95% CI 0.821–0.941). Weighted κ values varied from 0.581 to 0.908.

### 3.3 Construct and Criterion Validity

The Responsible Decisions Scale showed positive low‐to‐moderate correlations with self‐efficacy, satisfaction with life, and functional autonomy, and a negative correlation with an external locus of control. Conversely, the Self‐Neglected Decisions Scale correlated negatively, ranging from slight to moderate, with these variables, and positively with an external locus of control. The Independent Decisions Scale correlated slightly and negatively with self‐esteem, whereas the Guided Decisions Scale showed a slight positive correlation with life satisfaction (Supporting Information S1: Table 3). Regression analyses showed that the Responsible Decisions Scale was positively associated with self‐efficacy (β = 0.280, *p* < 0.001), life satisfaction (β = 0.166, *p* = 0.008), functional autonomy (β = 0.114, *p* = 0.034), and negatively with external locus of control (β = ‐0.140, *p* = 0.026), with small effects for all these predictors. Self‐esteem did not predict this style.

The Self‐Neglected Decisions Scale was negatively associated with self‐esteem (β = ‐0.371, *p* < 0.001) and positively with external locus of control (β = 0.227, *p* < 0.001), with moderate and small effects, respectively. The Guided Decisions Scale was significantly associated with greater life satisfaction (β = 0.218, *p* = 0.001) and higher external locus of control (β = 0.181, *p* < 0.008), with small effect sizes. Lastly, the Independent Decisions Scale was only positively associated with self‐efficacy (β = 0.211, *p* = 0.004), and negatively with self‐esteem (β = ‐0.169, *p* = 0.010), both showing small effect sizes (Table 4).

**TABLE 4:** Multiple regression analysis of the factor scores of the self‐care decision scales and psychological variables (*n* = 350).

	Independent Decisions Scale		Responsible Decisions scale		Self‐Neglected Decisions Scale		Guided Decisions Scale	
Scales	β	*p*	β	*p*	β	p	β	*p*
GSES	0.212	0.003	0.280	< 0.001	0.100	0.090	−0.022	0.763
SWLS	−0.049	0.462	0.166	0.008	−0.052	0.376	0.218	0.001
LCBS	−0.093	0.167	−0.140	0.026	0.227	< 0.001	0.181	0.008
RSES	−0.169	0.010	0.054	0.378	−0.371	< 0.001	0.012	0.856
IADL	0.018	0.761	0.114	0.034	−0.055	0.280	−0.053	0.359
R^2^	0.045		0.171		0.245		0.046	

Abbreviations: GSES, General Self‐Efficacy Scale; IADL, Instrumental Activities of Daily Living Index; LCBS, Locus of Control Behavior Scale; RSES, Rosenberg Self‐Esteem scale; SWLS, Satisfaction With Life Scale; β, Standardized regression coefficient.

The decision‐making styles were significantly associated with the three dimensions of the SCI, with effect sizes ranging from small to moderate. Specifically, the Independent Decisions Scale was negatively associated with Self‐Care Maintenance (β = ‐0.240, *p* < 0.001), Monitoring (β = ‐0.181, *p* < 0.001), and Management Scales (β = ‐0.166, *p* = 0.002), as was the Self‐Neglected Decisions Scale (β = ‐0.358, *p* < 0.001; β = ‐0.347, *p* < 0.001; β = ‐0.305 *p* < 0.001, respectively). In contrast, the Responsible Decisions Scale showed consistent positive associations across all three scales (β = 0.238, *p* < 0.001; β = 0.343, *p* < 0.001; β = 0.273, *p* < 0.001, respectively). Lastly, the Guided Decision Scale showed a small positive association only with Self‐Care Management Scale (β = 0.142, *p* = 0.008) (Table 5).

**TABLE 5:** Regression analysis of the three scales of self‐care inventory and factor scores of the self‐care decision‐making styles (*n* = 350).

Self‐care inventory scale	Self‐care maintenance		Self‐care monitoring		Self‐care management	
Decision‐making style scale	β	*p*	β	*p*	β	*p*
Independent	−0.240	< 0.001	−0.181	< 0.001	−0.166	0.002
Responsible	0.238	< 0.001	0.343	< 0.001	0.273	< 0.001
Self‐neglected	−0.358	< 0.001	−0.347	< 0.001	−0.305	< 0.001
Guided	0.102	0.056	0.050	0.335	0.142	0.008

Abbreviation: β, Standardized regression coefficients.

## 4 Discussion

This study aimed to develop and assess the psychometric properties of the DECIDE inventory. To the best of our knowledge, this is the first instrument specifically designed to evaluate self‐care decision‐making styles in older adults. The inventory comprises four distinct scales, each measured by five items: Independent, Responsible, Self‐Neglected, and Guided. These scales offer a simple, practical tool for assessing decision‐making styles relevant to self‐care in later life.

The findings support the dimensionality, construct validity, internal consistency, and stability of each scale. As hypothesized, associations with related constructs were largely confirmed, although some unexpected findings emerged. The responsible decisions style was associated with higher functional autonomy, life satisfaction, self‐efficacy, and an internal locus of control, with small but meaningful effect sizes. Among these predictors, self‐efficacy showed the largest effect, supporting the idea that greater confidence in one's abilities is linked to assuming responsibility for health decisions (Easom 2003). Interestingly, self‐esteem did not show a significant association with this style, possibly due to overlapping effects with self‐efficacy and life satisfaction or indirect influences mediated by these variables (Ryff 2014). Future studies should clarify these mechanisms. The self‐neglected decisions style was primarily associated with poor self‐worth and a perceived lack of personal control in health decisions, rather than with low autonomy, life dissatisfaction, or poor self‐confidence. Self‐esteem showed the greatest effect, suggesting that deficits in self‐worth may undermine decision‐making capacity more directly than contextual or situational factors. The independent decisions style was associated with higher self‐efficacy but, unexpectedly, lower self‐esteem, suggesting that confidence in self‐care abilities may coexist with a diminished global self‐view, potentially reflecting social isolation, limited external validation, or a compensatory reliance on personal capability. The guided decisions style was characterized by a positive life appraisal combined with a tendency to defer decisions, rather than lower self‐efficacy, self‐esteem or autonomy. Further research is needed to explore these unexpected patterns.

Our four decision‐making styles share similarities with those identified by Scott and Bruce (1995) in the General Decision‐Making Style Inventory (GDMS) and are consistent with the conceptual perspective proposed by Thunholm (2004), suggesting that they reflect broader, well‐established patterns of decision‐making. The responsible decisions style aligns with the ‘Rational style’, as both emphasize deliberate, confident, and autonomous decision‐making. The guided decisions style shares similarities with the ‘Dependent style’, reflecting a preference for guidance and reliance on others in the decision processes. The self‐neglected decisions style resembles the ‘Avoidant style’, although in this case decision avoidance appears to stem from low self‐worth and an external locus of control. Lastly, the independent decisions style echoes aspects of the ‘Intuitive style’ through reliance on personal judgments, but its association with lower self‐esteem suggests a nuance not fully captured by the GDMS. This comparison highlights that, while the two instruments share common ground, the decision‐making styles identified in our study may reflect age‐ and health‐specific variations. According to Thunholm (2004), such differences can be linked both to habitual decision‐making patterns and to basic cognitive processes, such as information processing, self‐evaluation and self‐regulation, which consistently influence response patterns across different decision‐making tasks and situations. Further investigation is warranted to explain their role in self‐care decision‐making.

We found that decision‐making styles were associated with different levels of self‐care. The responsible decisions style was associated with greater self‐care maintenance, monitoring, and management, supporting evidence that thoughtful, informed decisions enhance effective self‐care (Riegel et al. 2012). In contrast, the Independent and self‐neglected decisions styles were negatively associated with self‐care across all domains, suggesting that either making decisions without HCP guidance or disengaging from one's health decisions hinder effective self‐care (Riegel et al. 2012). Notably, the guided decisions style was associated only with self‐care management, indicating that reliance on HCPs may be particularly useful in managing complex or urgent health situations rather than in routine maintenance or monitoring tasks. These findings highlight the importance of decision‐making processes on self‐care and provide additional support for the validity of the DECIDE inventory.

### 4.1 Implications

In clinical practice, the DECIDE inventory could help nurses identify older adults' dominant self‐care decision styles and tailor interventions accordingly. For those with an independent decisions style, it is important to respect autonomy while providing reliable resources (e.g., educational materials, interactive apps, and evidence‐based tools) and foster trust in HCPs (Anshari et al. 2021). For the responsible decisions style, interventions should reinforce proactive behaviors through education, peer support, and access to health technologies (Lewy 2015). Older adults with a self‐neglected decisions style may benefit from strategies to improve motivation, self‐esteem and personal control, while addressing functional limitations and ensuring access to services (e.g., transportation, medications, daily living assistance). Those with a guided decisions style would benefit from clear instructions, regular follow‐ups, and integrated care approaches like case management to ensure care continuity (Briggs et al. 2018). The DECIDE inventory can be easily integrated into routine assessments, requiring minimal training and time, while offering valuable insights for personalized care. Intervention studies tailored to self‐care decision‐making styles are needed to confirm their effectiveness in enhancing self‐care and health outcomes.

### 4.2 Limitations

We acknowledge several limitations. First, the use of a convenience sample limits the generalizability of our findings. Although recruitment across multiple Italian regions increased sample diversity, broader and more representative sampling is needed. Second, the decision‐making styles identified in this study reflect those of the current cohort of older adults living in Western countries. These styles have been shaped by the distinctive social, educational, and economic conditions experienced by this generation, including lower levels of formal education, economic hardships, and periods of rapid industrialization. Future cohorts of older adults may exhibit different self‐care decision‐making styles. Consequently, our findings should be interpreted within the historical and cultural context of the studied population and future research should replicate this study in other generational and cultural settings. Third, not all psychometric properties of the DECIDE inventory were assessed in this study. Further testing is therefore needed to confirm its psychometric properties and applicability across diverse populations.

## 5 Conclusions

This study provided robust psychometric evidence supporting the DECIDE inventory, a new instrument developed to identify the dominant styles adopted by older adults when making decisions about their self‐care: Independent, Responsible, Neglected, and Guided Decisions Styles. Further validation studies are recommended to confirm its reliability, validity, and generalizability across diverse populations and settings. Establishing the DECIDE inventory's validity and reliability could enhance its utility in clinical practice, facilitating more personalized care strategies tailored to individual decision‐making preferences among older adults.

## Author Contributions

We confirm that all the authors made substantial contributions to the conception or design of the study, or to the acquisition, analysis, or interpretation of data, as follows: Maria Matarese, Maddalena De Maria, Barbara Riegel, and Marzia Lommi contributed to the study's conception and design, or to data acquisition, analysis, and interpretation. Maria Matarese, Maddalena De Maria, and Barbara Riegel were involved in drafting the manuscript or critically revising it for important intellectual content. All authors gave final approval of the version to be published.

## Ethics Statement

The study was approved by the Ethics Committee of the University Campus Bio‐Medico (No. 88/2021‐OSS). All procedures were performed in compliance with relevant laws and institutional guidelines and have been approved by the appropriate institutional committee. The privacy rights of human subjects have been observed and informed consent was obtained for experimentation with human subjects.

## Conflicts of Interest

The authors declare no conflicts of interest.

## Patient or Public Contribution

Older adults contributed to the study by providing feedback and interpreting the instrument items during the development phase.

## Supporting information


**Table S1:** Check list COSMIN.

## Data Availability

The data that support the findings of this study are available from the corresponding author upon reasonable request.
